# Domestic violence and abuse in local child safeguarding policy: How is the problem represented?

**DOI:** 10.1111/hsc.14086

**Published:** 2022-10-25

**Authors:** Alexander Russell, Keith Clements, Robbie Duschinsky, Emma Howarth, Tammy Mayes, Alma Reisel, Barry Coughlan

**Affiliations:** ^1^ School of Clinical Medicine, University of Cambridge Cambridge UK; ^2^ National Children's Bureau London UK; ^3^ Department of Public Health and Primary Care, Primary Care Unit, University of Cambridge Cambridge UK; ^4^ School of Psychology, University of East London London UK; ^5^ Lived experience, parent advocate & activist of numerous charities Guildford UK; ^6^ London Borough of Hackney London UK

**Keywords:** child welfare, domestic violence, policy analysis, safeguarding

## Abstract

Within the United Kingdom, domestic violence and abuse (DVA) is the most commonly identified factor within child in need assessments, with rates increasing in recent years in addition to ‘lockdown’‐related spikes. This article examines the representation of DVA in local child safeguarding policies using Bacchi's (2009) ‘What is the problem represented to be?’ approach. Policies were collected from the websites of all the child safeguarding partnerships of England in July 2021. In total, we identified 59 policies. These policies are designed to guide local responses to DVA across services and thus have potential for substantial impact on practice across health and social care. Our results suggest that local DVA policy in England exists within a conceptual framework which spotlights the individual and lacks attention to their context. We argue that these policies focus on adults, neglecting attention to children within their own safeguarding policies. This is through children being peripheralized within the conceptualisation of ‘victim’ and the assessed adult risk being used as a proxy measure for the risk to child. Demographic discussions build an image of DVA as an issue that can affect anyone, but with little acknowledgement of the vulnerabilities facing proportions of the population and their complexities – when such vulnerabilities are discussed, they are individualised and viewed in absence of their societal causes, potentially eclipsing critical elements of a child's experience of DVA. The implications of our results are wide‐ranging but suggest a need to refocus on children and their context within local DVA policy.

**What is known about this topic?**

- There are substantial differences between local authorities with regards to child safeguarding.
- It is difficult to assess the risk posed to children by domestic violence and abuse.
- Domestic violence is a heavily gendered issue, with other contextual adversities known to reinforce its impact.

**What this paper adds?**

- Local policy constructs domestic violence and abuse through a narrow lens; one which focuses on the individual in absence of their context.
- Policies focus on ‘incidents’ of domestic violence and abuse; a term which may not match the lived experience of children.
- Within a significant number of policies there is little local tailoring of policy text.

## 1 INTRODUCTION

Domestic violence and abuse (DVA) is the most common factor identified within assessment of ‘Children in Need’ in England with the proportion of these assessments identifying this as a factor increasing consistently from 40.6% in 2013/14 to 50.6% in 2018/19 (Department of Education, 2014, 2019). 1 This fits with similar patterns of increased DVA‐related crime, with decreasing charging rates (Cowling & Forsyth, 2021; Office for National Statistics, 2020) in recent years. During the initial Covid ‘lockdown’ period of April to June 2020, there were also known increases in the number of phone calls to national DVA phonelines (Office for National Statistics, 2020). Within England, DVA is a prominent concern, especially following the recent royal assent of the Domestic Abuse Act, which amongst many things defines children as victims of DVA for the first time (Domestic Abuse Act, 2021).

Policy is critical to the tackling of social issues; it sets out both the practical steps that should be followed in relation to an issue as well as setting the tone and bounds of public and professional discourse (Bacchi, 2009). Within policy‐making, there is a known gap between theory and practice as well as a need for improved evidence‐based policy (Clarke & Barwick, 2021; Hallsworth et al., 2011). It is, therefore, imperative that policy is not looked at superficially, but deeply examined for the ways in which it constructs problems and the way that this links to the utilisation of evidence within policy. Practically, the representations made within policy have tangible implications, as has been shown with alcohol and drug policy (Bacchi, 2018; De Kock, 2020; Lancaster et al., 2015), child mental health policy (Callaghan et al., 2017), gender inequality policy (Bacchi, 2016) and several others.

Prior to the royal assent of the Children and Social Work Act, 2017, the governance of local area safeguarding was organised into Local Safeguarding Children's Boards (LCSBs), convened by the local authority and consisting of a chair and members from relevant agencies specified in legislation (Children's Act, 2004). The 2017 Act abolished these boards and identified three local partners (local authority, local health service and police), with the authority to make their own ‘multi agency safeguarding arrangements’ to best exercise their functions (Wood, 2021). An emphasis of this change was to allow partners to foster a more flexible and localised approach to safeguarding, both in governance and function (Briggs & Harris, 2021; Department of Education, 2018). This was done in part to address some of the perceived issues associated with the board structure, such as uncertainty over their remit and purpose as well as resource limitations (Baginsky & Holmes, 2015). Whilst the local authority retains responsibility for social care interventions to safeguard children, the overall approach to safeguarding across local agencies, including the agreed thresholds for such intervention, is set out under the auspices of the multi‐agency safeguarding arrangement (Department for Education, 2018).

It is known that there are significant differences between different local authority responses with regards to child safeguarding, with the term ‘postcode lottery’ being commonplace within public discourse (Berg, 2021; Savage, 2018). The postcode lottery in children's social care is generally accepted to be damaging to children and their families. Previous examination of local threshold documents has shown that there is considerable variation for the initiation of both section 47 enquiries and early help measures in relation to DVA (Ellison & Renton, 2018).

Assessing the risk posed to children by DVA is difficult, with barriers identified relating to professional knowledge, skills and resources (Lewis et al., 2018; McTavish et al., 2019, 2020; Saxton et al., 2020; Turner et al., 2015). There is also a known lack of validated risk assessment tools for quantifying risk faced by adult victims and their children (CAADA, 2009; Edleson et al., 2007; Fitz‐Gibbon et al., 2019). It is also known that children are often not necessarily thought of as direct victims of DVA and that the dynamics of coercion and control are difficult to understand for those without lived experience of DVA (Lewis et al., 2018; Robinson et al., 2016, 2018; Saxton et al., 2020). The concept of coercive control is frequently only used with reference to the adult victim, with little acknowledgement of the significant impact that this has upon children, particularly when children are not involved in physical ‘incidents’ of abuse (Callaghan et al., 2018; Katz, 2016, 2020).

Thresholds within child safeguarding documents are often based on the assumption of a linear continuum of abuse, with the severity or future risk posed by the abuse corresponding with the degree of intervention required in terms of safeguarding. Whilst such an approach is easily understandable, it simplifies the various complexities that may be present within any given child's situation (Platt & Turney, 2014). These complexities are however, often not understood or appreciated at the ‘front‐door’ of the referrals process, where early categorisation of complex cases due to performance management pressures and other factors can result in neglect of wider challenges and adversities facing families (Broadhurst et al., 2010; Wilkins, 2015). This categorisation of cases itself then impacts upon actions taken, as when DVA is the reported concern, the social care intervention is less intensive, compared with other types of child maltreatment (Henry, 2018; Lawson, 2019; Victor et al., 2019).

Identification of DVA within a household is key to identifying children exposed to DVA. It is therefore prudent to examine the demographics of adults represented in these policies, as these affect a child's risk of experiencing DVA, the impact if it happens, and access to services. DVA is a heavily gendered crime, vastly impacting women over men (Feder & MacMillan, 2015), with a debate existing within academic literature as to how this is best represented within policy and law (Aldridge, 2021; Kelly & Westmarland, 2014; Kuskoff & Parsell, 2021). Additionally, there are other aspects of demographics that are of relevance to DVA, such as race, LGBTQIA+ identity, disability, old age and young age—all of which impact on the intersectional experiences of DVA that an individual may experience (Brownridge, 2006; Cannon & Buttell, 2015; Cho, 2012; Grossman & Lundy, 2007; Hughes et al., 2011; Policastro et al., 2015; West, 2012). These societal dynamics are known to affect both access and candidacy for services (Mackenzie et al., 2013), as well as potentiating the impacts of DVA (Grady et al., 2019). Nuance is required with regards to these issues, as people should not be singled out for unwarranted attention from social services based on their demographics, or have their relationships interpreted differently due to demographic risks – however, the implications for child outcomes are important.

Our research question was the following, ‘what is domestic violence and abuse represented to be in local child safeguarding policy?’. Using Bacchi's approach, we apply this question to the following four thematic areas: the conceptual framework of the policy documents; their characterisation of children's safeguarding needs; their attention to the contexts within which DVA occurs; and the perceived demography of parents involved in DVA.

## 2 MATERIALS AND METHODS

### 2.1 Definitions

There are two terms that require clarification of the reason for their use in this article. These are summarised below:

Firstly, this analysis uses the term ‘domestic violence and abuse’ (abbreviated as DVA)—a deliberately broad term which allows us to examine all members of a household that are impacted by DVA including children. This is also the terminology that is most commonly used in the policies that we have examined, although we note the recent use of the term ‘domestic abuse’ within the recent Domestic Abuse Act (2021) and the criticism of this framing for ignoring the gendered nature of DVA (Aldridge, 2021).

Secondly, we use the term ‘victim’ throughout our discussion—this is due to this being the most common language used within the policies we examined. It is not an apolitical term, and is one that has been appropriately criticised for its denial of agency to those impacted by DVA (Dunn, 2005). We note that we have not used the term ‘survivor’, which may be preferred by some. In using the term victim, we do so conscious of the tacit representations that this creates and explore these within the context of the policies analysed. We also use the term victim in the broadest sense, including both parents who experience DVA and children, emphasising the inclusion of the latter within the recent Domestic Abuse Act.

### 2.2 Design

**FIGURE 1:**
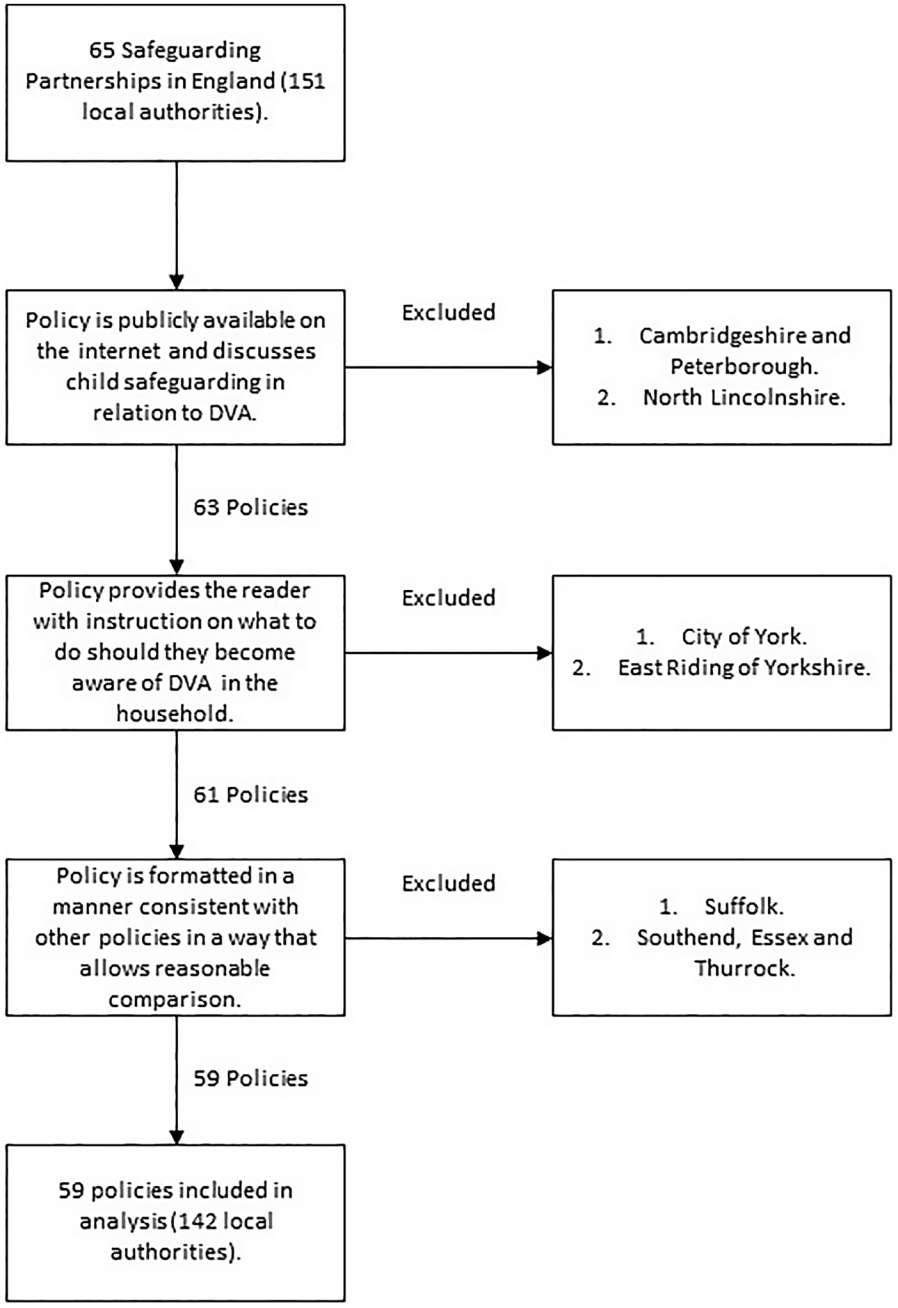
Inclusion criteria and excluded policies flowchart.

There are 151 local authorities responsible for the delivery of social care services for children in England (Ofsted, 2020), covered by 65 safeguarding partnerships. Fifty‐nine of these safeguarding partnerships had policies related to DVA that fitted our inclusion criteria, with these being collated in July 2021, and analysed from August to November 2021. Inclusion criteria are listed below, with full detail supplied in Figure 1.

1. Policy is publicly available on the internet and discusses child safeguarding in relation to DVA.
2. Policy provides the reader with instruction on what to do should they become aware of DVA in the household.
3. Policy is formatted in a manner consistent with other policies in a way that allows reasonable comparison.

Many safeguarding partnerships cover multiple local authorities, although most (69.5%) pertained to only one local authority. Others covered larger areas, such as the policies for London and Greater Manchester which covered 33 and 10 local authorities respectively. Where a safeguarding partnership covered multiple local authorities, the safeguarding partnership policy was analysed in preference to the local authority policy, if it was ascertainable. This was due to many of these local authorities not having policies that met inclusion criteria.

Textual analysis of the policies used Bacchi's (2009) ‘what is the problem represented to be?’ approach, which is centred around the following 6 questions:

1. What's the ‘problem’ represented to be in a specific policy or policies?
2. What presuppositions and assumptions underlie this representation of the ‘problem’?
3. How has this representation of the ‘problem’ come about?
4. What is left unproblematic in this problem representation? Where are the silences? Can the ‘problem’ be thought about differently?
5. What effects are produced by this representation of the ‘problem’?
6. How/where is this representation of the ‘problem’ produced, disseminated and defended? How could it be questioned, disrupted and replaced?

Once an initial overview took place, it became clear that a significant majority of policies used similar subheadings or content was similarly structured in some sections of the policies, and thus these sections were used to structure analysis. The names, content and prevalence of these sections are summarised in Table 1. Sections/content that featured in over 80% of the policies were taken forward as the focus for comparative qualitative analysis to generate themes using Bacchi's questions. Sections/content that were present in less than 30% of the policies were not used for the generation of themes but were examined for these themes once they had been generated.

**TABLE 1:** Prevalence of different sections/content amongst policies

Section/content	Number of policies	Percentage of policies
Introduction and definition	59	100
The risks of DVA, the impact of DVA, the signs of DVA and the barriers to disclosure	58	98.3
Enabling disclosure, issues surrounding DVA, and initial response to disclosure	58	98.3
Assessment, referral and protection and action to be taken	57	96.6
Clare's law, domestic violence prevention orders and the domestic violence disclosure scheme	53	89.8
Safety planning	13	22.0
Multi‐agency risk assessment conference	16	27.1
Appendices	14	23.7
Independent domestic violence advocates	2	3.9
Legal procedures	2	3.9
Child contact arrangements	2	3.9
Policies with sections not mirrored in any other policy	7	11.9

### 2.3 Analytic approach

Bacchi's questions were applied section by section to the policies to generate themes which could be focussed on during the study—these resulted in the sections that divide up the results and discussion of this paper. Due to the nature of this study focussing on local policy, we have focussed on answering these questions using the content of the policies and have only situated this within the wider context when it was necessary to do so. This has meant that certain questions are considered more fully than others—questions 1, 2, 4 and 5 are discussed at length, whereas questions 3 and 6 are only discussed to a limited degree. Detailed discussion of these latter two questions would require a historical account of the construction of these policies, as well as the current wider political context which is beyond the scope of this study.

Issues that were/were not mentioned within policies were coded in a binary manner; where it was appropriate to quantify the degree to which an issue was discussed, appropriate text was summated by means of manual identification and counting via Microsoft Word. The Monterey Language Services online word counter was then used to ascertain the total word count of each document to allow the length to which issues were discussed to be expressed as percentages of the whole policy. Following this, the advanced search function of Adobe Acrobat DC was used with all key terms to ensure all appropriate elements of text were counted.

## 3 RESULTS

### 3.1 Policy scrutiny

**FIGURE 2:**
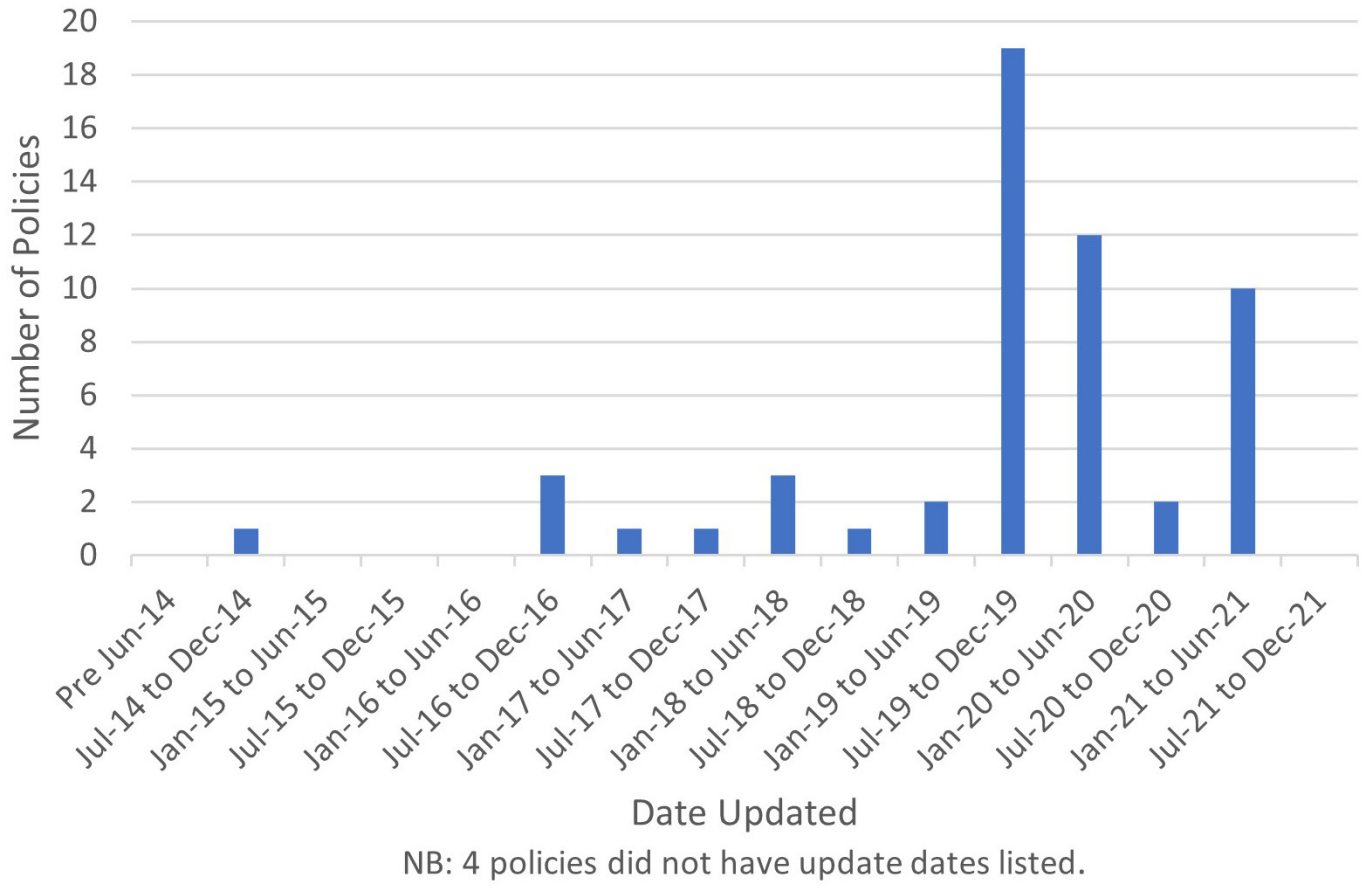
Distribution of dates that policies were last updated.

Policies varied in length (3 to 74 pages) and were last updated or reviewed (not stated in 4 policies) between September 2014 and July 2021, the interquartile range for these policy review dates was between September 2019 and March 2020. 11.9% of the policies had been updated between the royal assent of the Domestic Abuse Act in April 2021 and when these policies were collated in July 2021. The full distribution of dates when the policies were last updated is shown in Figure 2.

There was significant overlap between policy texts, with large sections of policies being identical across most safeguarding partnerships. Several child safeguarding partnership DVA leads were contacted about this finding—all responded that they were unaware of an urtext that this could be attributed to, but stated they thought that this was most likely due to the copying of text from other safeguarding partnerships. Searching online using elements of the identical text to attempt to find an origin document also yielded no results, suggesting that this is the most likely explanation. These common sections were coded, and the percentage of total word count they contributed to each policy calculated. This is summarised in Table 2.

**TABLE 2:** Distribution of common text amongst policies

Percentage of common text used in policies	Number of policies	Percentage of policies
0–20%	22	37.3%
20–65%	16	27.1%
65–85%	21	35.6%

The implication is that within a significant number of safeguarding policies, there is little to no local tailoring: text has been directly copied from other safeguarding partnerships' policies. This may be due to a desire for some standardised wording of local DVA policy or to copy best practice from elsewhere. Partnerships may also not feel able to develop their own tailored local policy, whether due to organisational pressures, a lack of DVA specific policy‐making expertise, or other factors.

These policies represent DVA within a conceptual framework that appears characterised by three core features.

1. First, there is a stark absence of attention to societal dynamics within these policies. The terms ageism, sexism, patriarchy, homophobia, transphobia and ableism do not appear in any policy. Racism is mentioned only in 10.1% of policies and the terms prejudice, discrimination and poverty are used in 8.5%, 6.8% and 6.8% of policies, respectively—this places the causative focus on the individual family, in absence of their context.
2. Secondly, these policies present DVA as something that occurs in ‘incidents’. The term appears repeatedly within policies (an average of 13.9 times per document), usually in referring to ‘an incident of DVA’. It was extremely rare to see any language that characterised DVA as something ongoing or pervasive, rather than localisable within discrete ‘incidents’.
3. Finally, all policies characterise DVA as a ‘problem’ that must be dealt with post‐occurrence. There is no mention of primary prevention of DVA across any of the policy documents. Whilst DVA prevention is not an explicit responsibility of safeguarding partnerships, the absence of its discussion reinforces it as something that occurs in ‘incidents’, requiring crisis management, rather than something requiring enduring change or prevention.

Two discursive features of the texts appeared prominent in hindering the reader in questioning these features of the conceptual framework: the way in which statistics are used and the presence of discernible errata within the text. Beginning with statistics, these policies use statistics to legitimate and naturalise the account offered of DVA. Yet they provide remarkably few references for the statistics they quote: 69.4% of policies have no clear referencing for any statistics quoted; those that did reference often did not for all their statistics; many of the references that were given were not able to be followed to the work from which the statistic was derived. Examples of these are found in Table 3.

**TABLE 3:** Statistics and the percentage of policies that quote them, for which a text accounting for them could not be identified

Statistic quoted	1/3 of DVA begins or worsens in pregnancy	Children witness ¾ of DVA incidents	Women experience on average 35 incidents before disclosing to the police
Percentage of policies that Include Statistic	67.8	23.7	11.9

The loose referencing of statistics suggests a casual approach to evidence, raising questions about the intended function of statistics in these documents. A further feature of the policy texts that suggests that the reader should not make comparison with external referents is that policies often have discernible errata present within their text, commonly manifesting as broken links, illegible text, or missing sections—in total 22.0% of policies had at least one of these. One broken link was to the ‘broken‐rainbow’ charity, an LGBT+ charity that ceased operations in June 2016. These broken links are usually to further sources of information, particularly local information—making the reader further reliant on the representations made within the policies themselves.

### 3.2 Safeguarding children

All policies reflected a ‘risk‐based approach’, with the use of Risk Assessments being a central theme of their content. The risk assessment tool used by the partnership is not stated in 35.6% of policies, despite these policies stating that professionals should familiarise themselves with these. The dominant risk‐assessment tool identified was the DASH Risk Assessment (CAADA, 2009), with 62.7% of policies stating that they use it, although it should be acknowledged that this tool is designed to assess risk to adults. The Barnardo's Domestic Violence Risk Identification Matrix (DVRIM) is recommended for use in addition to the DASH in 18.6% of policies to separately assess risk to children. Within 40.7% of policies, the DASH Risk Assessment is the only reference to DVA specific thresholds or referral criteria.

The criteria for referral to children's social care were outlined very differently across policies, with different methodologies being employed to explain criteria for referral, outlined in Table 4. In addition, Table 5 outlines specific criteria named in policies, which warranted consideration of a social care referral, regardless of the methodology employed to explain criteria for referral.

**TABLE 4:** Ways that policies outline referral criteria to children's social care

General referral criteria	Percentage of policies
Refer if concerned of ‘Significant Harm’ and/or link to the partnerships general thresholds or referral document; no DVA specific thresholds.	44.1
DVA‐specific thresholds of referral given within policy	45.8
Refer if Any DVA in Household	5.1
Refer if Barnardo's DVRIM shows threshold is met for referral	5.1

**TABLE 5:** Specific referral criteria to children's social care, irrespective of Table 4

	Pregnancy/child under 12 months	MARAC referral/ DASH 14+/high risk	Child made call/ injured/used as shield	Third incident in year
Percentage of policies that state to refer/consider referring	66.1	54.2	15.3	8.5

### 3.3 Representation of broader adversity as a context for domestic violence and abuse

The characterisation of the context of families affected by DVA shapes the construction of what kind of a problem DVA itself represents.

The issues of ‘mental health’, ‘substance abuse’ and ‘alcohol abuse’ are represented significantly in these policies, each appearing in over 90% of policies. 86.4% of the policies characterised these factors as reinforcing one another and increasing risk. These discussions were usually focussed on the parents, with mental health of children only being explicitly discussed in 22% of policies.

Honour‐Based Violence, Female Genital Mutilation and Forced Marriage are discussed in all policies—unsurprising as 98.4% of policies quoted the Home Office DVA (Home Office, 2013) definition which includes these forms of harm. An average of 2.2% of the policy texts are dedicated to discussion of these concerns, generally amalgamated as a single issue. 76.3% of policies spend over 1% of their word count discussing this, with 10.1% spending over 5% of their word count discussing this. Whilst this is not an entirely fair comparison, as these are forms of abuse closely related to DVA, rather than something represented as potentiating the impact of DVA, this still amounts to significant representation of these issues within DVA policy, compared with other possible aspects of contextual adversity such as poverty.

Coercive or controlling behaviour is not discussed in relation to children within any policy.

### 3.4 Gendering of language and parental demographic representation

#### 3.4.1 Gendering of language

93.2% of policies use gender‐neutral language regarding perpetrators and victims of DVA. The remaining policies either take an explicitly gendered approach (3.4%) or use mixed language, using different gendered approaches in different sections (3.4%). Notably one of the gendered policies is for London, and one of the mixed, Greater Manchester, which together cover 31.5% of local authorities in England and thus have particular bearing. Policies taking a gendered approach all carried a note at the beginning acknowledging that DVA could be perpetrated by women, and that these policies should be followed for all instances of DVA, regardless of gender. Within policies taking a gender‐neutral approach, often the only reflection of the gendered nature of DVA were within discussions of pregnancy.

In the gendered policies, the images of mothers as victims remained remarkably simple, discussing their vulnerability as women without contextualising this within patriarchy, sexism, or other societal dynamics. Notably, within these gendered policies, several representations come to the fore which use language that constructs victims as those failing the expectations of their gender, with diminished capacity to parent, with no reference to the perpetrator, their agency, or responsibility:

Quote 1: “… domestic [violence and] abuse may diminish a mother's capacity to protect her child/ren and mothers can become so preoccupied with their own survival within the relationship that they are unaware of the effect on their child/ren.”

Quote 2: “Mothers who experience domestic abuse are more likely to use prescription drugs, alcohol and illegal substances.”

(Quotes present within all gendered policies)

#### 3.4.2 Parental demographic representation

Different policies discuss different populations to different extents, usually in relation to a child's parents. The reason that this is included is that the recognition of DVA between parents is a potential first hurdle to the identification of children experiencing DVA. Therefore, it is important to see which types of parent are represented in these policies. The degree to which different demographics are represented in these policies is outlined in Table 6.

**TABLE 6:** Parental demographics and their representation in policies

Population	Young people (18–25)	Racial, cultural or immigration issues	LGBT+	Older people	Disability
Percentage of policies that mention	84.7	67.8	23.7	18.6	10.2
Mean number of words when discussed	192.6	87.1	103.3	139.5	71

Most policies take the approach of not specifically naming different demographics and their vulnerabilities. The exception is young people (18–25), who are discussed in the largest percentage of policies and to the greatest extent in terms of word count, relative to other demographics. There are two approaches that dominant policy frameworks take discussing risk to young people, either stating this is related to a vulnerability to Honour‐based violence (HBV), or by a more explorative section entitled ‘risk to teenagers’; the majority of which lists statistical information in prose about why this demographic may be more at risk of DVA.

The level to which racial, cultural and immigration issues are discussed within the policies can be framed in two ways, depending on whether one includes the following quote within the data, as it is the only reference to these issues in 42.4% of policies:

Quote 3: “Be alert to cultural issues when dealing with ethnic minority victims and that, in leaving a partner, they may be ostracised by family, friends and the wider community increasing the risks to their safety.”

(Quote present in 57.6% of policies)

Whilst this may be true, this is not a significant discussion of the effects that race, ethnicity or culture may have in relation to DVA; indeed, there is a conflation of culture with ethnicity in this, with no clarification as to what ‘cultural issues’ is intended to mean. There is also no discussion of the role that immigration issues may play within mechanisms that perpetrators may employ to control a victim.

A small minority of the policies discuss the additional vulnerabilities that LGBTQIA+ people may face when accessing DVA services, when this happens this usually is a discussion in relation to cisgender LGBTQIA+ people, with the only policies discussing transgender people being the two gendered policies. The quote discussing transgender people in both policies is the same:

Quote 4: “There are also issues around safe havens for transgender people and their children, and some women's refuges may not accept men who have not fully transitioned.”

(Quote present within all gendered policies)

This quote shows the narrow category of woman that is being portrayed within these. By using the term ‘men who have not fully transitioned’ over ‘transgender woman’, this language is actively excluding trans women from the category of victim.

Sections discussing disability and old age often associated and sometimes conflated the two issues, if they were discussed at all.

## 4 DISCUSSION

### 4.1 Policy scrutiny

Our results suggest that these safeguarding policies construct a conceptual framework that has several notable absences: namely their absence of societal dynamics, their post‐occurrence‐based approach, and their focus on ‘incidents’ of DVA. The presence of discernible errata and difficulties found when examining the statistics quoted create a representation that is difficult to question or investigate further. This conceptual framework has the effect of placing the causative focus on the individual and thus the opportunities for intervention examined are pitched at this level—similar frameworks have been found in recent child mental health policies, showing that this is not a DVA specific finding (Callaghan et al., 2017). These implicit emphases may have material impacts for practice, as has been shown with alcohol and drug policy (Bacchi, 2018; De Kock, 2020; Lancaster et al., 2015) and gender inequality policy (Bacchi, 2016). For instance, the use of the term ‘incident(s) of DVA’ is worth noting, as the temporal connotation of this is that DVA comes in bouts, with beginning, middle and end, with no abuse occurring between incidents. Such a linguistic framing has been argued to not match the experiences of victims, but to use the language of abusers (Kelly & Westmarland, 2016).

There is also a lack of local tailoring of a significant amount of the policies, with a common text being heavily used across policies. The implication of this is that there is a significant amount of copying of policies across partnerships—whilst this is not necessarily surprising or undesirable as it minimises the work that overly pressured safeguarding partnerships need to invest in generating or reviewing policy, it also appears to have contributed to the perpetuation of errata and unquestioned assumptions. This suggests a desire within safeguarding partnerships for the generation of some common text that could then be used within these policies, which might prove useful if such text is generated and scrutinised by professionals, experts by experience and academics.

The policies appear to show a casual attitude towards evidence, whilst displaying markers of evidence‐based policy such as extensive quoting of statistics. One possible explanation is that safeguarding partnerships and practitioners both anticipate that practitioners will not treat the claims of the policy documents as having much bearing for them; their existence is more a matter of compliance. Professionals are perhaps expected to instead rely on professional experience or training. Alternatively, by appearing as evidence‐based documents, one could argue that professionals are less likely to use their professional judgement, relying on these policies as justification for their actions. This said, it is known that it is difficult to understand the dynamics of coercion and control in DVA without lived experience, even for professionals (Robinson et al., 2018) and that this casual attitude towards evidence may be an element that maintains these misunderstandings.

### 4.2 Safeguarding children

Whilst the new Domestic Abuse Act now defines children as victims within law, an element of the Act lauded by various charities that operate in this area (Action for Children et al., 2020; Welsh Women's Aid, 2021; Women's Aid, 2021), this definition change has yet to be incorporated into local policy despite 13.6% of policies having been updated since the royal assent of the Act on 29th April 2021. Within all policies, therefore, the dominant view is that children are not conceptualised as ‘victims’ of domestic abuse per se, instead they are represented as being impacted indirectly via by others victimisation or as ‘collateral damage’. Whilst only a small proportion of the policies had been updated between the royal assent of the Domestic Abuse Act and the time of policy collation, the lack of adoption of this definition at the local level is notable. Follow up of this study will be required to see if this remains the case as further partnerships update their policies.

Our results have shown that within the 45.8% of policies that outline DVA specific thresholds for referral, the assumption of a linear continuum of abuse is typical (Platt & Turney, 2014). Policies that use the ‘common text’ for this section use a three‐level approach, with bullet points as to what might indicate different levels of risk and appropriate response to these. These lists of factors are DVA‐specific with little discussion of broader adversity or protective factors, narrowing down the range of pertinent information that may be included in a referral, or examined afterwards. This may then feed into a hasty and over‐narrow categorisation of and response to complex cases within children's social care (Broadhurst et al., 2010; Wilkins, 2015), further narrowing examination of broader adversity. The implication of this for children is a narrowing of the range of potential solutions proposed following assessment.

Within the 44.1% of policies that do not use DVA‐specific threshold criteria, the only reference to DVA specific assessment is through use of the DASH risk assessment, used in almost all of this type of policy. Yet the DASH risk assessment is a risk assessment for adult victims, and these are child safeguarding policies—thus, the only DVA specific measure within these is the risk posed to the adult, which is then used as a proxy measure for the risk to child. This is of particular concern when there have recently been questions raised about both the application and predictive accuracy of the DASH for adult victims (Robinson et al., 2016; Turner et al., 2019). One might suppose that a solution to this is to use pre‐existing child DVA risk assessments, however, there is lack of these (Edleson et al., 2007), with the Barnardo's DVRIM, used in 18.6% of policies, having never been subject to validity testing (Fitz‐Gibbon et al., 2019).

Within all policies, there is no mention of coercive and controlling behaviour in reference to children, despite research suggesting that this has a significant impact (Callaghan et al., 2018; Katz, 2016, 2020). It is also suggestive that only 22% of policies discuss the mental health of children. Previous research has found that referrals characterised using the term ‘incident’ are more likely to be taken up by children's social service (Platt, 2006). The physical focus of ‘incident’‐based terminology (Kelly & Westmarland, 2016) suggests that the language of ‘incident’ used in these policies may also direct attention towards physical abuse, whilst missing the significant non‐physical ‘hidden impact’ of DVA (James, 2020). Our impression is that there is a consistent theme of children's experiences being pushed to the periphery within their own safeguarding policies, through the lack of representation of their experiences as victims, a focus on physical forms of abuse, and an adult focussed assessment process.

### 4.3 Representation of broader adversity as a context for domestic violence and abuse

The most common aspects of broader adversity discussed within these policies are Female Genital Mutilation, Forced Marriage and Honour Based Violence—generally amalgamated as a single issue by the policies.

Other related issues discussed in policies were mental health issues, alcohol, and drug abuse, which policies represented as issues that reinforced eachother and increased risk. This is in line with wider discourse about these factors as a ‘toxic trio’, though the use of the term ‘toxic trio’ itself was rare in these policies. A lack of attention to other contextual adversities, for instance poverty, was notable across all the documents examined. These findings are potentially a reflection of wider research which either explicitly (Children's Commissioner, 2018b; Fuller‐Thomson et al., 2021) or implicitly (Burlaka et al., 2017; Children's Commissioner, 2018a) places emphasis on the ‘toxic trio’ factors. The priority given to these factors in these policies is concerning given their limited evidence base (Skinner et al., 2021) and a longstanding push within research to broaden our understanding of childhood adversity (Afifi et al., 2020; Felitti et al., 1998; Hood et al., 2021; Mersky et al., 2017).

### 4.4 Gendering of language and parental demographic representation

#### 4.4.1 Gendering of language

Our results show two things in relation to gendered language: firstly, there can be considerable room for local influence in the generation of these policies. It may be the safeguarding partnerships that cover the greatest number of local authorities who are able to take the conscious decision to step away from the dominant policy framework used within smaller partnerships.

Secondly, there is a large emphasis on gender neutral approaches to DVA within policies – perpetuating the idea that this is not a gendered issue. This fits within wider policy discourse and the current implicit emphasis from the UK Government who did not add clauses reflecting the gendered nature of DVA to the recent domestic abuse act, despite resistance from many organisations within the violence against women and girls (VAWG) sector (Aldridge, 2021). Gender neutral approaches to DVA suggest that this is an issue that impacts the genders equally, despite the empirical reality that women are at much greater risk (Feder & MacMillan, 2015); whilst we welcome that policies acknowledge that men can be victims of DVA – the significant impact that DVA has on women over men needs to be stated, particularly in relation to domestic homicide. In 2020, 77% of domestic homicide victims were female, with 96% of the suspects for these being male. Of the male victims, roughly half (47%) of the suspected perpetrators were female (Office for National Statistics, 2020).

Within the policies using gendered language, there was often no mention of the perpetrator or their responsibility in causing DVA, as evidenced in quotes 1 and 2. This use of passive constructions through the use of passive voice and nominalisation, hiding the agency and responsibility of perpetrators has been noted in academic papers discussing DVA (Lamb, 1991) and has also recently also been shown to be present within DVA policy of Queensland, Australia (Kuskoff & Parsell, 2021). These types of constructions are particularly used when policies are seeking to build an image of victims of DVA using statistics, with the statistics quoted in Table 3 entirely failing to reference the perpetrator as the agent of the acts. The result of this phraseology is to remove the perpetrator of the abuse from the representation of the issue—removing them from blame and hiding the gendered axis of DVA. This focus on the victim using language that implies they have failed to meet gendered and parental expectations implicitly blames them for the DVA that their children are experiencing. This implied inherent weakness ultimately focuses judgements on the caring abilities of the victim rather than the need to protect the children from the perpetrator.

#### 4.4.2 Parental demographic representation

There is a large representation of the risks posed to young people by DVA in their own relationships, compared with limited discussion of other demographics and their vulnerabilities. When discussion of these groups took place, this took place at the individual level, with no little to no references to how these are related to societal dynamics and various axes of oppression.

This is surprising, considering that it is known that there are considerable risks posed to these groups as well as barriers to accessing services. This has been shown in relation to racial minorities (Cho, 2012; Grossman & Lundy, 2007; West, 2012), LGBTQIA+ people (Cannon & Buttell, 2015; Donovan & Barnes, 2019, 2020; Donovan & Hester, 2010), disabled people (Brownridge, 2006; Hughes et al., 2011) and older people (Policastro et al., 2015). It is possible that these absences are partially a consequence of many of these policies being out of date and thus not being reflective of current thinking and practice.

The lack of representation of these demographics within DVA policy, constructs them as factors that are not critically related to DVA. The lack of acknowledgment of societal dynamics or axes of oppression and simplistic language used in relation to these demographics places a focus on the individual, in absence of their context. The emphasis on young people as an at‐risk demographic, in relative absence of discussion of other vulnerabilities means that DVA is primarily represented as a young person's issue, creating an archetypal image of whom a victim of DVA is. The implications of this are wide ranging, but for the purposes of this study, ultimately have the potential to exclude the needs of any children whose parents do not fit within this image of archetypal ‘victim’.

## 5 STRENGTHS AND LIMITATIONS

This study has the strengths of examining DVA through a lens that encompasses multiple perspectives across the whole of England, allowing insights into the representations of DVA that are generally accepted and those that are contested. It also comes at a pivotal time within DVA legislation and policy, with the recent passing of the Domestic Abuse Act, 2021, which most of these policies have yet to be updated to reflect. This is both a strength and a limitation as it offers a unique opportunity for academic input into local DVA policy, but also limits how much this study can comment on the impacts of the Act on local policy. In addition to this, this study examines only one of the arms by which the various representations of DVA in public discourse are set and maintained, which is reflected in this study's inability to address some of Bacchi's (2009) questions in detail. Finally, this study is a study of policy that has implications for practice, it is not a study of practice, thus the degree to which the representations discussed here manifest within practice requires its own empirical study.

## 6 CONCLUSIONS

Our results suggest that local DVA policy in England has to date been developed in the context of a narrow conceptual framework, focused on the individual and neglecting attention to their context. Children are side‐lined within their own safeguarding policies, being peripheralized within the conceptualisation of ‘victim’, with the risk to adult often being used as a proxy measure for the risk to the child. We would urge a need to recentre attention towards children in these policies, broaden the temporally limited ‘incident’‐based approach, and introduce greater attention to societal dynamics within policy frameworks. Doing so would change the problem perceived, and the kinds of intervention therefore implied. This might be achieved by greater alignment between policy and evidence and more deliberate approach to evidence within safeguarding policy documents.

## 7 RECOMMENDATIONS

### 7.1 For policy and practice

- Children should be referred to as victims of DVA within safeguarding policies.
- Societal power dynamics of racism, poverty, patriarchy, homophobia, etc, need to be incorporated into policy. These need to be discussed in relation to the risks posed to certain populations, how this may intensify the impact of DVA and create barriers to services. Discussion of demographics needs to recognise the potential biases that may lead to certain parents being singled out for attention from children's social care, to not reinforce existing axes of oppression.
- The emphasis on ‘incidents’ of DVA should be reconsidered. It is unlikely that this matches onto the experience of children, or the social reality of DVA.
- A ‘best‐practice’ template for universal sections of policy should be generated, which local areas can then adapt to their local context. This template should be adequately scrutinised by safeguarding professionals, experts by experience and academics.
- Policies should include references to all statistics quoted within documents to link research and policy together.

### 7.2 For research

- Appropriate evidence‐based tools for the assessment of children exposed to DVA need to be developed; these should be appropriately DVA specific but have the ability to incorporate broader adversity.
- The casual treatment of evidence in the policy documents may or may not reflect the seriousness with which practitioners are expected to treat safeguarding policy documentation. This would need to be examined empirically.
- Follow up of this study will be needed to establish whether the definitional change of children as ‘victims’ of DVA is adopted at the local level, following the passing of the Domestic Abuse Act.

## AUTHOR CONTRIBUTIONS

Dr Alexander Russell made all the necessary revisions throughout the publication process. Dr Russell also conducted the initial analysis and coding of the policies, as well as writing the first draft of the manuscript. This was discussed, edited and reformulated as necessary throughout with advice, guidance and support from Dr Robbie Duschinsky and Dr Barry Coughlan. Mr Keith Clements, Dr Emma Howarth, Mrs Tammy Mayes and Ms Alma Reisel all provided detailed insight and feedback on the manuscript from their personal, academic or professional experiences that was integral to the manuscript in its current form. All authors read and approved the final version of the manuscript.

## CONFLICT OF INTEREST

None.

## Data Availability

The data that support the findings of this study are openly available in at each safeguarding partnerships' respective website. The data that support the findings of this study are also available on request from the corresponding author.

## Endnote

1 Data for 2019/20 states that this is still the most common factor, but this statistic is now broken down into several categories, making the percentage incomparable (Department of Education, 2019).
